# Assessment and prevention of acute health effects of weather conditions in Europe, the PHEWE project: background, objectives, design

**DOI:** 10.1186/1476-069X-6-12

**Published:** 2007-04-24

**Authors:** Paola Michelozzi, Ursula Kirchmayer, Klea Katsouyanni, Annibale Biggeri, Glenn McGregor, Bettina Menne, Pavlos Kassomenos, Hugh Ross Anderson, Michela Baccini, Gabriele Accetta, Antonis Analytis, Tom Kosatsky

**Affiliations:** 1 Department of Epidemiology Local Health Authority Roma E, Rome, Italy; 2 Department of Hygiene & Epidemiology, University of Athens Medical School, Athens, Greece; 3 Department of Statistics, University of Florence, Florence, Italy; 4 Department of Geography, King's College London, London, UK; 5 WHO, Regional Office for Europe, Rome, Italy; 6 Department of Astrogeophysics, University of Joannina, Joannina, Greece; 7 Division of Community Health Sciences, St. George's, University of London, UK

## Abstract

**Background:**

The project "Assessment and prevention of acute health effects of weather conditions in Europe" (PHEWE) had the aim of assessing the association between weather conditions and acute health effects, during both warm and cold seasons in 16 European cities with widely differing climatic conditions and to provide information for public health policies.

**Methods:**

The PHEWE project was a three-year pan-European collaboration between epidemiologists, meteorologists and experts in public health. Meteorological, air pollution and mortality data from 16 cities and hospital admission data from 12 cities were available from 1990 to 2000. The short-term effect on mortality/morbidity was evaluated through city-specific and pooled time series analysis. The interaction between weather and air pollutants was evaluated and health impact assessments were performed to quantify the effect on the different populations. A heat/health watch warning system to predict oppressive weather conditions and alert the population was developed in a subgroup of cities and information on existing prevention policies and of adaptive strategies was gathered.

**Results:**

Main results were presented in a symposium at the conference of the International Society of Environmental Epidemiology in Paris on September 6^th ^2006 and will be published as scientific articles. The present article introduces the project and includes a description of the database and the framework of the applied methodology.

**Conclusion:**

The PHEWE project offers the opportunity to investigate the relationship between temperature and mortality in 16 European cities, representing a wide range of climatic, socio-demographic and cultural characteristics; the use of a standardized methodology allows for direct comparison between cities.

## Background

Interest in the impact of weather on human health is increasing, especially in the light of potential climate changes. The rapid increase of greenhouse gases in the atmosphere is expected to increase both mean temperature and temperature variability around the world [1,2]. Climatologists project that, in temperate climates, a 2–3°C increase in average summer temperatures will double the frequency of periods characterized by extremely high temperatures [3]. Such changes would be characterized by the increased frequency and intensity of heat waves and cold spells, and could lead to an increase in heat related illness episodes and deaths.

The association between high and low temperatures and mortality has been investigated in several studies. Most investigations have focused on the effect of temperature on health through a time series approaches or focusing on extreme events (heat waves, cold spells) using episode analysis.

A review of the epidemiological studies on the effect of high temperatures on mortality conducted after 1970 identified sensitive groups among the elderly, persons with pre-existing cardiovascular and respiratory diseases and/or those of low socioeconomic status [4]. The heat effect appears within few days of exposure and some harvesting is observed [4,5]. The heterogeneity of the impact of heat on health reflects geographical, climatic and cultural variability, as well as different capacities to adapt to extreme temperatures and needs to be addressed more in depth in a large scale study [4-7]. Recently, studies on the effect of high temperatures on morbidity provided evidence of an increase in emergency hospital admissions for specific causes in young children and subjects over 75 years of age, though with a smaller effect for admissions than for mortality [8]. Schwartz et al. reported an effect of heat on cause-specific admissions within a few days after exposure and a short-term displacement of the events (harvesting effect) [9].

With regards to the effects of cold, several studies have suggested that there is a considerable burden in terms of mortality, especially in the elderly and in persons affected by chronic respiratory or cardiovascular diseases [5,7,10-12]. This effect is generally more prolonged than for heat-related deaths [10-13] and, in contrast with evidence during heat waves, extreme cold seems not to be followed by any mortality displacement [13]. The impact on morbidity usually anticipates death as suggested by the biological mechanisms underlying cold-related illnesses [14]. However, the influence of cold on outcomes other than mortality has, to date, received little investigation. The few studies present have focused on cardiovascular admissions and results showed an association with low temperatures [9].

In the 1990s, the APHEA (Air Pollution and Health: an European Approach) project investigated the short-term effects of air pollution on health throughout Europe [15]. In the time series analysis performed for this project, temperature was considered an important confounder of the association between air pollution and mortality [16].

The project "Assessment and Prevention of Acute Health Effects and Weather Conditions in Europe" (PHEWE) was initiated in 2002, with the general aim of assessing the association between weather and acute health effects (daily mortality and hospital admissions) in Europe and to provide information for public health policy on preventive and adaptive actions. The specific objectives of the project were:

- to create a European database of meteorological variables and health indicators;

- to perform time series analysis using a standardized methodology and evaluate the short term health effects of weather conditions on daily mortality and daily hospital admissions, both during warm and cold seasons;

- to investigate the role of air pollution as a potential effect-modifier using a standardized methodology;

- to define a synoptic weather classification system in different European areas, and to evaluate synoptic categories associated with increased mortality and morbidity;

- to experiment the use of Heat/health watch warning systems (HHWWS) to predict in advance high risk weather conditions;

- to produce a health impact assessments of weather conditions on mortality;

- to define public health actions aimed at the prevention of adverse health effects of weather in Europe.

## Methods

### The database

Data was collected for 16 European cities, representing a large spectrum of climatic conditions: Athens, Barcelona, Budapest, Krakow, Dublin, Helsinki, Ljubljana, London, Milan, Paris, Prague, Rome, Stockholm, Turin, Valencia, and Zurich, including about 30 million European citizens (Table 1). The number of city residents ranges between 6,8 million (London) to 260.000 (Ljubljana), with the percentage of the elderly population (over 75 years) between 5% (Helsinki) and 10% (Barcelona). The gender and age-adjusted mortality rate is highest in Budapest (1093.6) and lowest in Milan (439.4).

**Table 1:** PHEWE cities: characteristics and data series available

	**Characteristics**			**Health data – Time series used**	
		
**City**	Population	% 75+	Mortaliy rate (× 100.000)*	Mortality	Hospital Admissions
Athens	3 188 305	6.4	663.6	1992–1996	
Barcelona	1 512 971	10.1	542.5	1992–2000	1994–1997
Budapest	1 797 222	7.3	1093.6	1992–2001	1997–2000
Dublin	481 854	5.3	826.7	1990–2000	1994–2001
Helsinki	955 143	5.0	590.0	1990–2000	
Krakow	741 510	8.9**	805.4	1990–1996	
Ljubljana	263 290	5.9	719.1	1992–1999	1997–1999
London	6 796 900	6.8	584.5	1992–2000	1992–2000
Milan	1 304 942	9.5	439.4	1990–2000	1990–1999
Paris	6 161 393	6.1	554.0	1991–1998	1991–1995
Prague	1 183 900	7.0	779.4	1992–2000	
Rome	2 812 573	7.3	497.4	1992–2000	1998–2000
Stockholm	1 173 183	8.5	576.2	1990–2000	1990–2000
Turin	901 010	9.2	479.5	1991–1999	1995–1999
Valencia	739 004	7.6	595.6	1995–2000	1996–2000
Zurich	990 000	n.a.	n.a.	1990–1996	1990–1996

* adjusted for gender and age

** >= 70 years

#### Health data

Mortality data were provided from 16 cities and hospital admission data from 12 cities for all available years in the period 1990–2000 (Table 1), all referring to the city residents and to events that occurred in the city, except for Dublin, where hospital admissions referred also to non-residents. Taking into account the results of previous studies [10,12,17] and the biological plausibility of the health effects [7,14,18,19], the following causes of death and hospital admissions were selected for all ages combined and specific age groups (0–14 yrs, 15–64 yrs, 65–74 yrs, 75+ yrs): all causes (excluding external causes), ICD-9: 1–799; cardiovascular diseases, ICD-9: 390–459; cerebrovascular diseases, ICD-9: 430–438; respiratory diseases, ICD-9: 460–519.

Data from cold and warm seasons were analyzed separately, defining summer as the months from April to September and winter as the months between October and March.

Table 2 gives an overview of the mean daily number of deaths by cause and season. Data on cerebrovascular mortality were not available in three cities (Athens, Paris, Zurich). For all other causes the mean daily counts were lower in the summer season.

**Table 2:** Mortality data: daily mean and standard deviation (sd) by cause and season.

	Total mortality (ICD-IX < 800.0)				Cardiovascular (ICD-IX: 390–459)				Cerebrovascular (ICD-IX: 430–438)				Respiratory (ICD-IX:460–519)			
			
	Summer*		Winter**		Summer*		Winter**		Summer*		Winter**		Summer*		Winter**	
			
	mean	*sd*	mean	*sd*	mean	*sd*	mean	*sd*	mean	*sd*	mean	*sd*	mean	*sd*	mean	*sd*
Athens	67.46	10.79	78.26	13.18	32.62	7.58	39.46	8.4	-	-	-	-	4.19	2.21	5.08	2.57
Barcelona	35.89	6.58	42.75	9.17	12.96	3.96	16.58	5.11	3.61	2.01	4.45	2.07	3.08	1.88	4.71	3.07
Budapest	70.99	11.01	79.82	13.38	34.89	7.32	40.9	8.92	70.97	11	79.8	13.36	2.22	1.6	3.12	2.24
Dublin	11.42	3.45	13.62	4.12	4.91	2.19	5.98	2.57	0.99	1.01	1.23	1.1	11.42	1.25	2.12	1.68
Helsinki	17.05	4.18	18.45	4.6	8.23	2.96	8.81	3.1	2.42	1.56	2.66	1.65	1.37	1.18	1.79	1.47
Ljubljana	6.29	2.58	7.06	2.89	2.55	1.63	3.03	1.84	0.63	0.8	0.73	0.86	0.36	0.62	0.53	0.75
London	149.01	16.45	179.23	29.35	60.96	10.51	73.5	13.62	14.14	4.2	16.91	4.71	23.73	6	36.58	15.79
Milan	26.3	5.87	31.86	7.17	9.85	3.41	13.21	4.22	2.88	1.78	3.58	1.97	1.65	1.38	2.54	1.93
Paris	115.69	13.84	128.17	16.44	34.81	6.57	40.01	7.91	-	-	-	-	7.69	3.06	10.33	4.15
Prague	34.94	6.51	38.52	7.49	20.27	4.87	22.66	5.62	5.64	2.62	6.19	2.72	1.12	1.09	1.53	1.32
Rome	52.81	9.55	61.71	10.48	20.92	5.52	26.62	6.46	4.87	2.36	5.79	2.6	2.58	1.72	3.73	2.52
Stockholm	27.85	5.41	30.81	6.3	13.47	3.84	14.93	4.28	2.96	1.72	3.34	1.81	2.15	1.5	2.76	1.88
Turin	19.14	4.65	23.18	5.35	7.91	3.01	10.3	3.49	2.71	1.67	3.34	1.9	1.02	1.04	1.62	1.44
Valencia	14.64	4.11	17.92	5.27	5.28	2.41	6.97	2.94	1.57	1.27	1.91	1.46	1.37	1.2	2.09	1.76
Zurich	11.63	3.52	13.53	3.84	5.24	2.36	6.31	2.59	-	-	-	-	0.6	0.79	1.01	1.09

note: * April–September, ** October–March

Emergency hospital admission records for the same causes (plus influenza, ICD-9: 487) were selected, following the APHEA-2 project protocol [15]. Only main causes of admission were considered. In table 3 mean daily admission counts are summarized by cause and season. Two cities could not provide all requested causes, namely Paris and Zurich. Generally, admission counts were higher in the cold season, especially for respiratory diseases.

**Table 3:** Hospital admission data: daily mean and standard deviation (sd) by cause and season.

	**Cardiovascular ***(ICD-IX: 390–459)*				**Cerebrovascular ***(ICD-IX: 430–438)*				**Respiratory ***(ICD-IX:460–519)*			
			
**City**	Summer*		Winter**		Summer*		Winter**		Summer*		Winter**	
						
	mean	*sd*	mean	*sd*	mean	*sd*	mean	*sd*	mean	*sd*	mean	*sd*
Barcelona	21.8	*6.0*	25.9	*6.3*	5.0	*2.3*	5.6	*2.4*	15.9	*5.3*	23.5	*7.6*
Budapest	110.8	*50.1*	119.1	*50.0*	14.5	*7.5*	15.2	*7.6*	25.7	*10.0*	37.0	*15.3*
Dublin	25.9	*6.4*	27.3	*6.7*	5.1	*2.4*	5.5	*2.4*	22.6	*5.7*	30.3	*10.4*
Ljubljana	11.2	*4.9*	12.4	*5.4*	1.5	*1.2*	1.6	*1.3*	6.7	*4.4*	8.2	*4.6*
London	163.8	*31.8*	171.2	*34.7*	28.3	*6.8*	30.0	*7.5*	125.2	*25.3*	178.6	*52.7*
Milan	71.2	*26.3*	81.6	*24.7*	14.0	*5.1*	15.2	*4.9*	25.6	*10.4*	34.4	*11.1*
Paris	126.5	*45.3*	146.5	*44.3*	n.a.	*n.a.*	n.a.	*n.a.*	59.0	*19.4*	85.0	*22.7*
Rome	120.3	*27.0*	133.6	*26.1*	25.1	*6.1*	26.7	*6.0*	43.1	*11.4*	63.2	*18.3*
Stockholm	48.0	*12.1*	50.8	*12.2*	10.4	*3.6*	10.9	*3.7*	18.3	*6.1*	24.2	*7.9*
Turin	25.2	*6.4*	28.5	*6.8*	7.2	*2.8*	7.7	*2.9*	10.1	*4.0*	15.9	*6.4*
Valencia	12.4	*4.1*	13.6	*4.6*	3.1	*1.8*	3.2	*1.9*	9.2	*3.6*	14.4	*5.6*
Zurich	8.2	*3.4*	9.1	*4.9*	n.a.	*n.a.*	n.a.	*n.a.*	n.a.	*n.a.*	n.a.	*n.a.*

* April–September, ** October–March

#### Meteorological variables

For each city, data were retrieved from a weather station located in the city centre as well as from the nearest airport weather station for the entire study period. The following meteorological variables, recorded every three hours, were collected: air temperature, dew point temperature, wind speed, wind direction, sea level pressure, total cloud cover, solar radiation, precipitation, visibility. Quality control included a descriptive overview of the variables in all cities, detecting possible errors and extreme values, testing for homogeneity and correcting erroneous values where possible.

Table 4 gives an overview of the daily mean values observed in the participating cities. Mean summer temperatures ranged between 12.0°C in Helsinki and 23.5°C in Athens, in Valencia the lowest inner-city temperature range was reported. Relative humidity levels were highest in Dublin (81%) and lowest in Athens (57%). In winter, the lowest temperature was observed in Helsinki (-0.9°C) and the highest in Valencia (13.7°C), which is also the city with lowest relative humidity (69%) and the smallest temperature range. In the last column of the table, the values for maximum apparent temperature (see exposure assessment) are included. For Barcelona, 3-hourly meteorological data were not available; hence maximum apparent temperature could not be calculated and mean apparent temperature was used instead. As expected, the reported values are lower than those of other cities with similar climatic conditions (Valencia, Athens, Rome).

**Table 4:** Meteorological data: daily mean values (mean, minimum and maximum)

Summer period (April–September)															
	**temperature (°C)**			**relative humidity (%)**			**sea level pressure (hPa)**			**wind speed (m/s)**			**max apparent temperature (°C)**		

**CITY**	**mean**	**min**	**max**	**mean**	**min**	**max**	**mean**	**min**	**max**	**mean**	**min**	**max**	**mean**	**min**	**max**

Athens	23.5	7.6	34.3	57	23	89	1013.0	945.8	1031.3	3.3	0.3	11.6	27.9	7.9	41.6
Barcelona	21.7	8.6	34.2	66	29	99	1015.2	993.7	1030.6	6.7	0.9	22.3	23.3 *	6.5*	36.9*
Budapest	18.2	1.5	29.8	61	30	97	1014.5	992.6	1033.6	2.6	0.7	8.8	21.9	0.2	38.8
Dublin	12.5	1.4	21.0	81	53	100	1014.8	974.7	1038.5	4.7	1.1	12.0	14.7	1.5	28.5
Helsinki	12.0	-6.5	25.4	71	28	98	1012.7	982.9	1035.6	3.6	0.9	9.6	14.3	-3.7	32.8
Krakow	15.0	-1.6	26.6	77	45	98	1015.7	991.1	1033.1	2.3	0.0	10.1	19.1	-2.3	35.8
Ljubljana	15.9	0.6	26.5	75	33	98	970.8	949.2	985.4	1.6	0.2	7.0	20.1	-1.7	35.4
London	15.1	3.2	28.0	71	42	96	1015.5	984.1	1036.3	3.4	0.7	9.3	18.1	1.5	35.2
Milan	20.0	2.5	29.4	72	26	100	1014.2	991.5	1031.9	1.7	0.0	9.4	25.4	2.7	40.8
Paris	16.1	2.1	30.2	72	32	100	1015.9	988.1	1032.9	4.0	1.0	11.9	19.7	1.5	39.4
Prague	15.1	-1.7	28.7	70	31	98	972.5	947.0	990.4	3.8	0.3	12.0	17.8	-3.3	36.3
Rome	20.5	6.1	30.3	72	25	94	1014.2	992.6	1031.8	3.1	0.5	12.5	26.1	5.9	40.5
Stockholm	12.8	-3.2	26.6	72	36	99	1012.9	985.6	1036.1	3.3	0.6	8.1	15.4	-2.1	34.0
Turin	18.5	3.0	27.9	74	32	97	1014.2	993.0	1032.0	1.4	0.0	7.7	23.4	4.2	45.8
Valencia	22.3	10.5	30.0	66	32	92	1015.1	995.1	1030.9	3.3	1.1	9.9	29.5	10.6	44.9
Zurich	15.1	1.4	26.2	73	42	97	1016.4	993.2	1034.6	2.1	0.2	6.0	19.0	0.7	35.2

Winter period (October–March)															

	**temperature (°C)**			**relative humidity (%)**			**sea level pressure (hPa)**			**wind speed (m/s)**			**max apparent temperature (°C)**		

**CITY**	**mean**	**min**	**max**	**mean**	**min**	**max**	**mean**	**min**	**max**	**mean**	**min**	**max**	**mean**	**min**	**max**

Athens	13.1	0.7	26.5	70	35	92	1018.2	988.9	1035.7	3.2	0.0	12.8	14.8	0.8	34.1
Barcelona	13.4	1.9	25.2	69	37	100	1018.3	990.2	1038.2	7.0	0.0	22.1	12.3*	0.2*	27.9*
Budapest	4.0	-12.1	19.5	77	36	100	1019.7	988.9	1045.7	2.6	0.5	8.6	5.0	-9.7	25.7
Dublin	7.0	-4.2	17.8	85	54	100	1012.2	971.1	1046.3	6.0	0.6	17.5	7.5	-4.6	19.8
Helsinki	-0.9	-24.1	14.7	85	44	99	1010.8	949.5	1054.4	4.1	0.1	11.5	-0.7	-14.5	14.8
Krakow	2.3	-21.4	17.9	86	53	100	1019.2	987.7	1046.9	2.8	0.0	11.5	3.3	-14.4	25.4
Ljubljana	3.0	-13.3	18.4	83	26	100	973.5	939.8	995.5	1.3	0.0	8.0	4.4	-9.9	24.3
London	7.5	-5.2	18.9	81	52	100	1016.1	978.6	1044.3	3.7	0.4	12.5	8.4	-5.6	24.5
Milan	7.1	-6.5	22.7	81	20	100	1019.1	986.8	1041.0	1.3	0.0	9.4	8.8	-6.3	32.6
Paris	6.9	-10.7	20.0	84	37	100	1018.1	983.3	1044.8	4.8	0.5	14.6	7.7	-9.2	25.4
Prague	2.5	-19.7	19.0	84	42	100	973.8	974.5	996.8	4.6	0.0	16.3	2.6	-13.4	22.5
Rome	10.5	-2.0	25.8	80	34	98	1017.1	990.4	1039.1	3.2	0.3	13.1	13.5	-1.0	41.5
Stockholm	1.3	-16.7	15.8	85	44	99	1010.9	947.9	1049.1	3.7	0.3	10.3	1.3	-12.9	19.4
Turin	6.0	-6.9	20.2	76	25	99	1019.5	989.2	1041.4	1.1	0.0	9.5	8.1	-5.9	29.5
Valencia	13.7	3.4	26.0	69	29	98	1018.7	993.5	1034.8	3.1	0.3	12.2	18.1	3.2	35.9
Zurich	4.0	-11.7	17.0	82	44	98	1020.7	987.2	1044.6	2.4	0.5	10.3	4.8	-9.2	23.5

* for Barcelona the mean apparent temperature is reported

#### Exposure assessment

Previous studies have used a variety of exposure measures, including maximum, minimum or average temperature, apparent temperature, humidity and dew point temperature but to date there is no standard indicator of heat or cold stress [5,13,20-22]. In the present study, maximum apparent temperature (Tappmax) was chosen as the exposure variable, which is an index of thermal discomfort based on air temperature and dew point temperature [23]. Tappmax is defined as the highest value of the 3-hourly apparent temperature values, using the following formula:

AT = -2.653 + 0.994 Temp + 0.0153 (Dew)^2^

where AT is apparent temperature, Temp is the air temperature in °C and Dew is the dew point temperature in °C [24,25].

#### Air pollution data

The data collected within the APHEA-2 project were updated and integrated according the project's procedures [15]. The following pollutant measurements were recorded at each monitoring station (maximum 6): SO_2_(mean 24-hours), TSP or Black Smoke (mean 24-hours), PM10- if available-(mean 24-hours), NO_2 _(maximum 1 hour, mean 24-hours), O_3 _(maximum 1 hour, maximum 8-hours moving average) CO (maximum 8-hour moving average).

The selection of the monitors was based on local criteria, mainly on the completeness of measurements and representation of population exposure. A standardized procedure was used to fill-in days with missing data [15].

#### Other variables

Through a complementary questionnaire information on other confounders and potential effect modifiers was gathered from each city, such as holidays and unusual events during the study period (e.g. health services or transportation strikes, floods, earthquakes), percentage of households with air conditioning facilities and annual restrictions on home heating use. Questions regarding the cities' population, and details concerning data quality were also included, which were useful in order to characterize the different city populations (Table 1) and necessary to complete the Health Impact Assessment.

#### City-specific analysis

For the city-specific analyses a Generalized Equation Estimation (GEE) approach was proposed as an extension of Generalized Linear Models to analyze longitudinal data, as the observations on different subjects (clusters) can be assumed independent and the observations on the same subject correlated [26].

For each city, there was an outcome variable (number of deaths or hospital admissions) and several covariates (confounders, apparent temperature, other meteorological variables), observed on different days. A marginal Poisson distribution of the dependent variable and correlation between observations during one summer/winter were assumed, while observations from different summer/winter periods were considered independent.

An exploratory analysis was carried out in order to identify the appropriate dependence structure for each season to be used in the GEE, and was similar to the approach described in Chiogna and Gaetan [27,28]. This approach is based on dynamic regression models, combined with a genetic algorithm for the semi-automatic selection of the best model over a large model space, covering different specifications of the correlation structure within clusters and different specifications of the systematic components of the model. The model space included models with lagged values of apparent temperature, sea level pressure (up to lag 20), and an air pollutant (up to lag 5). For the error term, up to an ARMA (2,2) structure was specified. The idea was to highlight the common features across cities, in order to identify the most appropriate and common correlation structure within each season. The results of the exploratory analysis suggested to use a first order autocorrelation structure both for mortality and hospital admissions.

The common model applied to single city analysis took into account potential confounding effects of holidays, day of the week, seasonality and long-term time trend, barometric pressure, wind speed and air pollution levels, all modeled in parametric terms. An indicator of influenza epidemics was included in the model for cold season analyses (except for respiratory causes) [29]. Models for hospital admission analysis in the warm season included the moving average of total admission counts (ICD 9: <800) to offset population reduction during the summer holidays.

#### Exposure modeling

Based on the results of previous studies, the maximum apparent temperature of current and previous 3 days (lag 0–3) for the warm season and lag 0–15 for the cold season was chosen as the indicator of exposure; the delayed effect of the exposure was further investigated by distributed lag models in a sensitivity analysis [13].

The shape of the exposure-response curve between apparent temperature and log mortality/hospital admission rate was investigated, with a flexible approach, introducing a cubic regression spline for apparent temperature into the model.

For mortality, during the warm season a "turning point" or "threshold" was identified. The effect of high temperatures on summer mortality was also investigated focusing on the slope above the city-specific threshold of the exposure-response curve. City-specific thresholds were obtained *a priori *by a maximum likelihood approach, treating the apparent temperature corresponding to the minimum of the exposure-response curve as an unknown parameter [30].

For hospital admissions the effect of high temperatures in summer was investigated using a dummy variable for maximum apparent temperatures above the 90^th ^percentile.

The delayed effect of exposure on health outcomes was investigated using constrained and unconstrained distributed-lag models that simultaneously included variables for the same day and up to 5, 10, 15, 20, 25, 30 and (for hospital admissions) 40 days.

The impact of high temperatures on mortality was investigated through a health risk assessment analyses.

#### Pooled analysis

In the second stage, the city-specific effect estimates were combined to obtain pooled estimates. Overall exposure-response curves were obtained through a fixed effect meta-analytical approach using the pooled data set and through a second stage meta-analytical approach [31], while the city-specific effect estimates and the city-specific curves for distributed-lag and time-varying effects were pooled by a hierarchical Bayesian modelling approach.

To reduce heterogeneity, pooled results were obtained grouping the cities, according to an *a priori *defined meteorological and geographical criteria, distinguishing between Mediterranean cities and North-Continental cities.

Second stage models including potential effect modifiers as covariates were applied in order to explore heterogeneity. Such effect modifiers included variables on the climate, the health of the population, and on air pollution levels and the correlation between air pollution concentrations and the meteorological variables.

#### Confounding and synergistic effect of meteorological and air pollution variables

Based on the models defined in the city-specific and pooled analysis, further exploration of the confounding effects of air pollutants was carried out. Possible effect modification of the impact of temperature on mortality by air pollutant levels was investigated using meta-regression models.

## Discussion

While previous studies focused on single cities, the present project investigated the health impact of weather on a large scale through a variety of climatic conditions and of socio-economic and demographic characteristics, applying the same methodology, thus allowing for comparison between cities and the pooling of results.

Previous studies showed that the temperature level corresponding to the minimum mortality level varies from city to city and across different latitudes according to the local climate and probably reflecting adaptation by the local population to the temperature range in both the hot and cold season [11,12,22,32-35]. The analysis of the heterogeneity of the effect in European areas was accounted for in the present project, describing city-specific "change points" of the dose-response relationship and the specific shape of the dose-response curves. In the pooled analysis, heterogeneity was reduced grouping the cities according to *a priori *geographical and meteorological characteristics.

Few studies have examined the effect of heat on outcomes other than mortality. In Chicago, during the July 1995 heat wave an 11% increase in hospital admissions was observed, with 35% of the increase among patients over 65 years [36]. More recently, studies performed in London and 12 US cities reported an increase of admissions for specific causes in the elderly and evidence of a harvesting effect [8,9]. To date, the present study is the largest one to investigate weather and hospital admissions in Europe.

In the present study, the role of meteorological variables other than temperature was investigated, assuming that they may contribute to the negative health effects. Therefore, an exposure indicator including dew point temperature was chosen for the time series analysis [23,25], and the excess mortality/morbidity associated with specific air masses was explored using a climatologic classification based on synoptic indexes [37-39]. The choice of maximum apparent temperature as the exposure variable for the time series analysis was driven by the fact that it comprises temperature and dew point temperature in a single parameter. The simplified formula for this indicator was considered the most suitable one on the basis of data availability and quality. Several other indexes of thermal discomfort have been proposed in meteorological literature [40-43]. Among these, apparent temperature has been used as exposure measure in recent studies assessing the heat effect [20,25,44-48]. The importance of using heat stress indicators that combine temperature and a measure of humidity is due to the fact that on hot days the degree of humidity influences the body's ability to cool itself by evaporation and perspiration. From a modeling point of view, given that apparent temperature is obtained as linear combination of temperature and square dew point temperature, there is a strong correspondence between a regression model where a linear term for apparent temperature is included and a model where a linear term for air temperature plus a quadratic term for dew point temperature are introduced. Therefore, the percent variation in mortality/morbidity associated to 1°C increase in apparent temperature is expected to be similar to that of a model adjusting for dew point temperature.

When comparing the results between cities it is important to consider that the use of mean apparent temperature for Barcelona led to lower threshold values and an underestimation of the effect. Furthermore, regarding the pooled estimates, the combination of results obtained using different exposure indicators increases heterogeneity and the risk of bias in the meta-analytic estimates, in particular when thresholds or exposure-response curves are combined. Therefore, sensitivity analyses was performed excluding Barcelona from the meta-analysis and no significant differences were found.

This project focused on time series rather than on heat wave episodes, using an approach, that has been successfully used in the analyses of the effects of air pollution. Such methods have the advantage that the population under study serves as its own control, and covariates that vary between subjects, but not over time, are not potential confounders [49,50].

Most time series studies performed so far showed an immediate effect of heat on mortality, with the maximum impact within two or three days (lag 0–3) [5,10-13,34], while for the cold season, an effect of up to fifteen days has been observed [10,12,13]. Knowledge on the lag time between exposure to extreme weather conditions and negative health outcomes is important for the development and planning of prevention plans by public health authorities and health care providers.

Evidence that the increase in mortality is followed by a deficit that, partly compensates the negative effect (harvesting) is contradictory [51]. In the present study the heterogeneity of mortality/morbidity displacement patterns between cities was systematically investigated.

There is much disagreement in literature concerning human acclimatization to changing weather [37,52,53]. While the issue was examined by comparing the threshold temperature in different geographic locations, the possibility of a short-term acclimatization was also evaluated by comparing the dose-response function in the first period of the summer with the effects modeled in the later part of the season. This allowed also for comparison of the impact of the first heat wave in one summer with the following ones.

Given the small number of events (mortality and admissions), a unique definition of winter and summer season was chosen in order to reach a reasonable statistical power, and sensitivity analysis was performed, focusing on the three central summer months (June–August).

The relationship between increase in air pollution levels and acute health effects has been well described in the USA and in Europe [50,16]. The levels of some of the pollutants associated with increase in mortality and hospital admissions are higher during the summer period in many European areas. A synergistic effect of warm temperature and air pollution on mortality has been suggested from time series analysis conducted in Athens, whereas no effect modification detected in a study in Philadelphia, USA [54,55]. The present study investigated the independent effect of meteorological variables of that of ambient air pollution, and explored whether there is synergy between the two factors.

The results of the mortality analysis were also used for the development of experimental Heat/Health Watch Warning Systems (HHWWS) in five cities (Rome, Barcelona, London, Paris, Budapest). An air-mass-based climatologic index was developed, applying a synoptic approach. Using meteorological forecast data, these models are able to predict the arrival of an oppressive air mass 72 hours in advance. Such warning systems have been successfully implemented in the United States [38], whereas in Europe before the 2003 heat-wave, only in Rome a HHWWS was experimented and implemented. Therefore, the pilot study in five PHEWE cities represents an important innovation in the field of heat health prevention. The development of a standardized protocol allows for knowledge and technology transfer to other European cities in the future.

The usefulness of early warning systems is closely linked to public health strategies aiming at the prevention of negative health effects of heat. In the present project an overview of existing prevention programs in the participating cities was obtained through a questionnaire. Moreover, physiological and behavioral adaptation measures, experiences with different HHWWS, urban planning, housing standards, and socio-economic determinants of vulnerability were summarized in a comprehensive literature review. The quantification of the effect of heat/air masses exposures in the different populations was addressed through a health impact assessment (years-of-life-lost approach). These results will contribute to policy development, public health decision-making, and will be an important input for cost-benefit analysis and risk communication. Guidelines for preventive strategies and health care actions taken to lessen morbidity and mortality effects can then be based on evidences arising from this project, namely the literature review, the investigation of the state-of-the-art in the participating cities (feasibility), and the identification of susceptible populations.

## Conclusion

The PHEWE project offers the opportunity to investigate the relationship between temperature and mortality in 16 European cities, representing a wide range of climatic, socio-demographic and cultural characteristics; the use of a standardized methodology allows for direct comparison between cities. The analysis of the effect of weather on hospital admissions in 12 cities is an innovation in Europe. The evidence arising from the project's results, namely the literature review, the investigation of the state-of-the-art in the participating cities (feasibility), and the identification of susceptible populations (target groups), offer an important contribution for guidelines for preventive strategies and health care actions taken to lessen morbidity and mortality effects. The results of this project contribute to policy development, public health decision-making, and will be an important input for cost-benefit analysis and risk communication.

## Competing interests

The author(s) declare that they have no competing interests.

## Authors' contributions

PM coordinated the project, supervised hospital admissions analysis and participated in all methodological discussions. UK coordinated the data collection, dealt with administrative and financial issues, organized the meetings and contributed to the writing of the present paper. KK was the leader of the Epidemiology and Statistics working group, shared the methodological decisions with PM and AB, supervised the analysis on winter mortality and was responsible for the investigation of air pollutants as potential confounders or effect modifiers. AB shared the development of the methodology with PM and KK and supervised summer mortality analysis. GMG was responsible for the development of HHWWS in five cities and was the leader of the Meteorology working group. BM was responsible for all public health related issues and the organisation of the final work shop. PK carried out the analysis of meteorological indicators for all cities. HRA was the leader of the Public Health working group. MB and AA participated in the methodological discussion and carried out mortality analysis for summer and winter, respectively. GA contributed to the methodological discussion and carried out hospital admission analysis. TK performed health impact assessment of heat on mortality in collaboration with AB and MB and developed and implemented the city specific questionnaire for the assessment of prevention programmes in place. All authors read and approved the final manuscript.
